# Genome Wide Identification of Mutational Hotspots in the Apicomplexan Parasite *Neospora caninum* and the Implications for Virulence

**DOI:** 10.1093/gbe/evy188

**Published:** 2018-08-25

**Authors:** Larissa Calarco, Joel Barratt, John Ellis

**Affiliations:** School of Life Sciences, University of Technology Sydney, New South Wales, Australia

**Keywords:** variant analysis, SNP hotspot, transcriptomics, nonsynonymous mutations, population structure

## Abstract

*Neospora caninum* is an apicomplexan parasite responsible for neosporosis, a disease causing hind limb paralysis in dogs and abortion in cattle, resulting in substantial economic losses to beef and dairy industries. Marked differences in pathogenicity exist between *N. caninum* strains suggesting that intrinsic genetic differences exist between them. These differences likely exist in genes expressed during the tachyzoite lifecycle stage which is responsible for the pathogenesis of neosporosis. An improved understanding of these genetic differences is essential to understanding *N. caninum* virulence, though such knowledge is scarce. Using a variant detection workflow we compared the tachyzoite transcriptomes of two *N. caninum* strains with different virulence properties: NC-Liverpool (virulent) and NC-Nowra (avirulent). This workflow identified 3130 SNPs and 6123 indels between the strains, and nine markers capturing 30 variants were Sanger sequenced for both strains. Sequencing of these loci was extended to an additional eight strains and subsequent phylogenetic analysis supported a genetic population structure comprised of two major clades with no geographical segregation. Sequence polymorphisms within coding regions of tachyzoite-associated genes were concentrated on chromosomes XI and XII, with 19 distinct tachyzoite-associated SNP hotspot regions identified within coding regions of the *N. caninum* nuclear genome. The variants were predominantly located in loci associated with protein binding, protein–protein interactions, transcription, and translation. Furthermore, 468 nonsynonymous SNPs identified within protein-coding genes were associated with protein kinase activity, protein binding, protein phosphorylation, and proteolysis. This work may implicate these processes and the specific proteins involved as novel effectors of *N. caninum* tachyzoite virulence.

## Introduction

*Neospora caninum* is a cyst forming coccidian of the phylum Apicomplexa first described as the cause of a potentially fatal neurological disease of dogs (Dubey et al. 1988a). However, its economic importance is primarily due to its role as the etiological agent of bovine neosporosis, a reproductive disease characterized by abortion and stillbirths in cows that is recognized as the leading global cause of bovine reproductive failure (Dubey and Lindsay 1996; Dubey 1999; Reichel and Ellis 2002; Dubey and Dubey 2003; Dubey et al. 2006; Reichel et al. 2007). Bovine infections with *N. caninum* have been reported in the Americas, Europe, Australia, and New Zealand, causing losses within the range of US $1.1 million in New Zealand, to an average total of US $546.3 million in the USA (Reichel et al. 2013). The combined annual losses due to *N. caninum* for the Australian and New Zealand dairy and beef industries are estimated to be greater than AU $110 million annually (Miller et al. 2002; Reichel and Ellis 2002).

*Neospora caninum* is a diverse species and several strains have been characterized revealing notable genotypic and phenotypic differences (Al-Qassab et al. 2010b). For example, the highly virulent NC-Liverpool strain causes foetal death in cattle (Atkinson et al. 1999), whereas the NC-Nowra strain has been evaluated for use as a live attenuated vaccine against bovine neosporosis, based on its low virulence in mouse models (Miller et al. 2002; Williams et al. 2007; Weber et al. 2013). NC-Liverpool infection in mice causes severe neosporosis, characterized by encephalitis, hind limb paralysis, and severe weight loss, whereas a Swedish bovine isolate, NC-SweB1, induces similar but significantly milder symptoms in a smaller number of infected mice (Atkinson et al. 1999). The NC1 strain of *N. caninum* is known to induce severe clinical manifestations including fetal death in cattle as well as polyradiculoneuritis and granulomatous polymyositis in infected dogs (Dubey et al. 1988a, 1988b, 1992; Innes et al. 2001; Maley et al. 2003). However, while there are studies that report marked differences in pathogenicity between *N. caninum* strains in mouse models, there are limited results published comparing the behavior of various strains in cattle. A study focusing on correlating fetal loss with *N. caninum* infection reported fetal death in pregnant heifers following inoculation of the BPA1 isolate at 118 days gestation (Barr et al. 1994). Furthermore, an absence of fetal death was reported in pregnant heifers inoculated with NC-Spain 1 H, an isolate of low virulence, whereas fetal death occurred in heifers inoculated with the control strain NC1 (Rojo-Montejo et al. 2009). Differences have also been demonstrated between virulent isolates NC-Spain 7 and NC1 in cattle, with respect to the timing of fetal death and immunological response, where NC-Spain 7 resulted in higher fetal mortality rates and an earlier and higher anti-*N. caninum* IgG response (Caspe et al. 2012).

These phenotypic differences reflect a genetically diverse species. Analysis of mini- and microsatellite repeats for over 100 *N. caninum* strains has revealed extensive genetic diversity (Regidor-Cerrillo et al. 2006, 2013; Basso et al. 2009; Al-Qassab et al. 2010a). A typing method based on randomly amplified polymorphic DNA (RAPD) resolved several *N. caninum* isolates into six genotypes (Schock et al. 2001). Additionally, Regidor-Cerrillo et al. (2006) performed multilocus microsatellite analysis of nine cultured *N. caninum* isolates with varying host ranges and geographical locations, which revealed distinct genetic profiles for the 12 microsatellite markers investigated. Similarly, a multiplex PCR targeting three microsatellites and three minisatellites (Tand-3, Tand-12, Tand-13, Cont-6, Cont-14, and Cont-16), was developed by typing 25 cultured *N. caninum* isolates which identified 11 genotypes (Al-Qassab et al. 2010a). Although these methods reflect the diversity of *N. caninum* as a species, they are based on repetitive sequences that are generally noncoding and their impact on parasite phenotype is unknown.

The Apicomplexa have evolved several unique features that aid them in their intracellular parasitic lifestyle. These include molecules that facilitate motility, host cell adhesion, and invasion. Apicomplexan parasites manipulate host cells through secretion of effector proteins produced by specialized secretory organelles unique to this phylum; micronemes, rhoptries, and dense granules (English et al. 2015). Micronemal (*MIC*) proteins are released upon contact with host cells and facilitate adhesion (Cerede et al. 2005), where for example *MIC2* plays a role in host-cell attachment, motility, and invasion in *T. gondii* (Lovett et al. 2000; Huynh and Carruthers 2006), and *MIC1* and *MIC3* are soluble adhesins Naguleswaran et al. 2001; Keller et al. 2002; Cerede et al. 2005). Rhoptry family proteins are then secreted into the host cell cytosol facilitating formation of the tight junction between the invading parasite and target host cell, culminating in the formation of the parasitophorous vacuole (Talevich and Kannan 2013). Shortly after host cell invasion, the dense granules release *GRA* proteins that may be involved in nutrient acquisition (Nam 2009; Leineweber et al. 2017). Studies of the closely related apicomplexan parasite *Toxoplasma gondii* have identified a range of virulence factors that exist as orthologues in *N. caninum*, including dense granule protein *GRA9* (Leineweber et al. 2017), *ROP5* (Reese et al. 2011; Ma et al. 2017b), and *ROP16* and *ROP18* (Saeij et al. 2006; Taylor et al. 2006; Lei et al. 2014; Ma et al. 2017a).

Although current typing approaches for *N. caninum* have confirmed genetic variation in repetitive elements, there is a lack of knowledge on polymorphisms occurring in the coding regions of its genome. Sequence polymorphisms within many notable virulence factors have been described in *T. gondii*. For example, the identification of sequence polymorphisms within *GRA6* and *GRA7* of *T. gondii* led to the development of serotyping technology that is now commonly used for genotyping strains within this species (Kong et al. 2003; Sousa et al. 2009). Similarly, differences in virulence properties reported between *N. caninum* strains might imply that genetic diversity exists within, upstream, or downstream of genes that are transcriptionally active in tachyzoites which are the life cycle stage responsible for the pathogenesis of neosporosis.

The present study employed a variant detection workflow to compare the transcriptomes of two *N. caninum* strains with markedly different virulence properties: NC-Liverpool (virulent) and NC-Nowra (avirulent in mice). Phylogenetic analysis of sequenced polymorphic markers identified in silico, revealed a population structure consisting of two major clades showing no obvious geographical segregation. Tachyzoite-associated polymorphisms were associated with kinase activity, ATP binding, protein–protein interactions, and proteolysis, implicating several proteins involved in these processes as potentially novel determinants of *N. caninum* virulence.

## Materials and Methods

### Parasite Culture for Nucleic Acid Extraction and Sequencing

*Neospora caninum* strains (supplementary file S1, table S1, Supplementary Material online) were grown in vitro using Vero cells as the host cell line, at 37°C in RPMI media supplemented with 10% heat inactivated horse serum. Total RNA was extracted from the tachyzoites using TriSure reagent (Bioline) and treated with RNAase-free DNAase (Sigma). RNA-seq was performed on three biological replicates of mRNA, each extracted from difference passages of both NC-Liverpool and NC-Nowra only. For each strain, two technical replicates (RNA-seq libraries) were prepared, constituting six libraries in total. The sequencing reads were generated using Illumina HiSeq2000, 100 base paired-end sequencing.

For laboratory confirmation of the SNPs, genomic DNA was also extracted using the solvent extraction technique, from cultured tachyzoites of *N. caninum* and *Neospora hughesi* strain NE1 (imported from ATCC). Briefly, cells were pelleted and then extracted three times with equal volumes of phenol and chloroform and then once more with chloroform only, with thorough vortexing and centrifuging at 13,000 × g for 1 min between extraction steps. The DNA was precipitated from the final aqueous phase by isopropanol and resuspended in 100 μl of ddH2O. The DNA extracts were stored at −20 °C until required.

### Read Quality Control and Mapping

Illumina reads were trimmed for quality and length using the Filter FASTQ tool (Blankenberg et al. 2010a), available on the Galaxy Platform (Blankenberg et al. 2010b) through the Garvan Institute for Medical Research (http://galaxyproject.org/; Last accessed June 2015). Illumina reads <15 base pairs long, and with per base quality scores <20, were discarded using the Filter FASTQ tool (Bao et al. 2014; Pabinger et al. 2014; Broad Institute 2015). A Perl script (supplementary file S1, Supplementary Material online) was used to ensure paired read information was preserved, resulting in two paired read files, and an unpaired (singlet) read file. These processed reads were next mapped to the *N. caninum* reference genome available from ToxoDB (NC-Liverpool genome, version 28, http://www.toxodb.org/toxo/; Last Accessed April 2018) using TopHat version 2.1.1 (Bao et al. 2014; Pabinger et al. 2014; Broad Institute 2015). Read mapping was optimized by adjusting alignment parameters to increase the overall read alignment rate, as detailed in supplementary file S1, Supplementary Material online.

### De Novo Transcriptome Assembly

An in-house reference transcriptome was created for NC-Liverpool by performing a de novo transcriptome assembly. The TopHat alignment tool was first used to map the *N. caninum* reads to the Vero genome, resulting in unmapped BAM files containing *N. caninum* reads that were sorted and converted into fastq files using scripts provided in supplementary file S1, Supplementary Material online. The resulting fastq files were assembled using Trinity (version 2.5.1) (Grabherr et al. 2011). Removal of redundant contigs was performed using CD-HIT-EST which sorts comparable nucleotide sequences based on a user-defined similarity threshold, and reports the longest sequence in each cluster as the representative contig (version 4.6.6) (Li and Godzik 2006). This step was included to ensure the same variants were not identified and duplicated in the final callset, within redundant contigs generated by Trinity from the same or very similar sequence reads. The scripts available in the Trinity package and the TransRate software package (version 1.0.3) (Smith-Unna et al. 2016) were employed to assess the quality of both the original and new transcriptome assembly following CD-HIT-EST analysis, using the parameters and thresholds provided in supplementary file S1, Supplementary Material online, where a similarity threshold of 0.8–0.85 was used (*n* = 5). A summary of assembly metrics assessed is contained within supplementary file S1, table S2, Supplementary Material online, and the NC-Liverpool transcriptome can be found in supplementary file S2, Supplementary Material online, in FASTA format. The NC-Liverpool transcriptome generated in-house was compared with published NC-Liverpool reference transcripts from ToxoDB by mapping RNA-seq reads generated in-house from NC-Liverpool. Variant calling was performed from the resulting BAM (binary alignment/map) files.

### Variant Calling

SAMtools (Li et al. 2009) was used to sort and index the “mapped” BAM files generated by TopHat, and to generate an mpileup (multi-sample pileup) output. This data was then imported into VarScan 2 (Koboldt et al. 2013) for variant calling using the recommended parameters. The identified variants were filtered using VarScan’s accessory scripts, which remove variants that do not meet thresholds pertaining to strand bias, sequence and variant coverage thresholds, mismatch quality sum, and read position bias. A detailed description of this workflow can be found in supplementary file S1, Supplementary Material online. Variants were visualized using the Integrative Genomics Viewer (IGV) (Robinson et al. 2011; Thorvaldsdottir et al. 2013). Briefly, the *N. caninum* reference genome FASTA file was uploaded to IGV (version 2.3.67), along with the sorted BAM files for each sample. Hundreds of variants were randomly selected for viewing from the SNP and indel lists produced by VarScan, and a set of high confidence variants were selected from amongst these for laboratory validation.

### Variant Annotation

The de novo transcriptome assembly generated for NC-Liverpool was queried against the published NC-Liverpool reference genome using a BLASTN search (version 2.7.1), to facilitate assignment of each transcript to a chromosome. High confidence hits (*E*-value ≤ 1*E*^−50^, Bit-score ≥ 200, and PID ≥ 90%) were subsequently cross-referenced with the contig location of each SNP, for allocation of SNPs to a chromosome. A BLASTX search was also performed querying the de novo NC-Liverpool transcriptome assembly against NC-Liverpool annotated proteins from ToxoDB (NcaninumLIV, version 30), to assign SNPs to a gene ID (PID ≥ 90%). The location of SNPs along the *N. caninum* genome was then plotted to investigate the distribution of variants. Circos plots (Krzywinski et al. 2009) were generated to present the SNP data set in the context of the *N. caninum* genome, and to determine whether any particular regions might represent mutational (i.e., SNP) hotspots. A gene region was classified as a mutational hotspot if it contained >15 SNPs within a 50 kb window. The genes within these regions and their SNP information were extracted for gene ontology analysis.

### Functional Analysis of Mutational Hotspots

InterProScan (version 68.0) was used to assign functional information to proteins putatively encoded by the genes within each SNP hotspot (Quevillon et al. 2005; Finn et al. 2017). For each of these hotspot genes, their respective protein sequences were analyzed using TMHMM (Sonnhammer et al. 1998), Phobius (Kall et al. 2004), and Philius (Reynolds et al. 2008), to identify potential transmembrane helices and signal peptides. The transcripts were also ranked by SNP density (contig length/number of SNPs) to investigate whether genomic regions with either a high or low SNP density were functionally significant. To do this, contigs were ranked on SNP density and contigs from within the first and third quartiles were extracted and their chromosome locations were identified. A *z*-test was performed to elucidate whether any chromosomes encoded a significantly larger number of SNP-dense contigs (*P* value <0.05). DNAPlotter (Carver et al. 2009) was then used to visualise the main features for chromosomes of interest, using available NC-Liverpool GenBank records (Ramaprasad et al. 2015).

### Identifying Nonsynonymous and Synonymous Mutations

A de novo transcriptome was generated for NC-Nowra using Trinity, as described previously for NC-Liverpool. TransDecoder (Haas et al. 2013) was used to identify candidate protein-coding regions within the transcripts based on nucleotide composition and open reading frame (ORF) length. The protein sequences generated by TransDecoder for NC-Nowra were subjected to a BLASTP search (PID ≥80%) against the protein sequences generated for NC-Liverpool via the same procedure, to identify transcripts with identical ORFs, and those with mismatches or gaps between the two strains. These two lists were then cross referenced against the list of SNPs identified by VarScan, to identify nonsynonymous and synonymous mutations. The protein sequences from transcripts found to contain nonsynonymous SNPs were subsequently submitted to InterProScan for functional annotation, and the elucidation of gene ontologies, domains, repeats, and protein superfamilies.

### Polymerase Chain Reaction (PCR) and Sanger Sequencing

PCR primers were designed to capture randomly selected variants identified by VarScan (supplementary file S1, tables S3 and S4, Supplementary Material online). All PCRs were prepared using the reagents provided in a MyTaq (Bioline) PCR kit. Each reaction contained 10 μM of each forward and reverse primer, 0.5 μl of MyTaq DNA Polymerase (5 U/μl), 2 μl of DNA template, and 5 μl of 5× MyTaq reaction buffer in a total volume of 50 μl. Each reaction was accompanied by a negative control, where DNA template was substituted with ddH_2_O. The temperature cycling conditions employed were as follows: 1) 95 °C for 5 min, 2) 95 °C for 1 min, 3) 57–61 °C (primer dependent—see supplementary file S1, tables S3 and S4, Supplementary Material online) for 40 s, and 4) 72 °C for 40 s. Steps 2–4 were repeated 39 times, followed by a final extension step (5) of 72 °C for 5 min. PCR was performed on genomic DNA extracted from cultures of NC-Liverpool and NC-Nowra, as well as NC1, JPA1, NC-SweB1, WA-K9, NC-Beef, BPA1, BPA6, and an additional NC-Liverpool that had been cryogenically stored since 1998. This NC-Liverpool passage from 1998 was included as a control to investigate the genetic stability of this isolate over several years. The PCR products were then subject to electrophoresis on 2% agarose gels containing GelRed, visualized under UV light, and excised from gels using a sterile scalpel blade. Amplicons were purified from gel slices using a Qiagen QIAquick Gel Extraction Kit in accordance with the manufacturer’s instructions. Sequencing was performed twice in both the forward and reverse direction on an ABI capillary sequencer, by the service provider Macrogen (South Korea). The ABI files were analyzed using SeqTrace (Stucky 2012). The forward and reverse sequences were assembled into contigs using an online version of CAP3 (Huang and Madan 1999). The resulting contigs were aligned for comparison using Clustal Omega (Sievers and Higgins 2014).

**Fig. 1.**
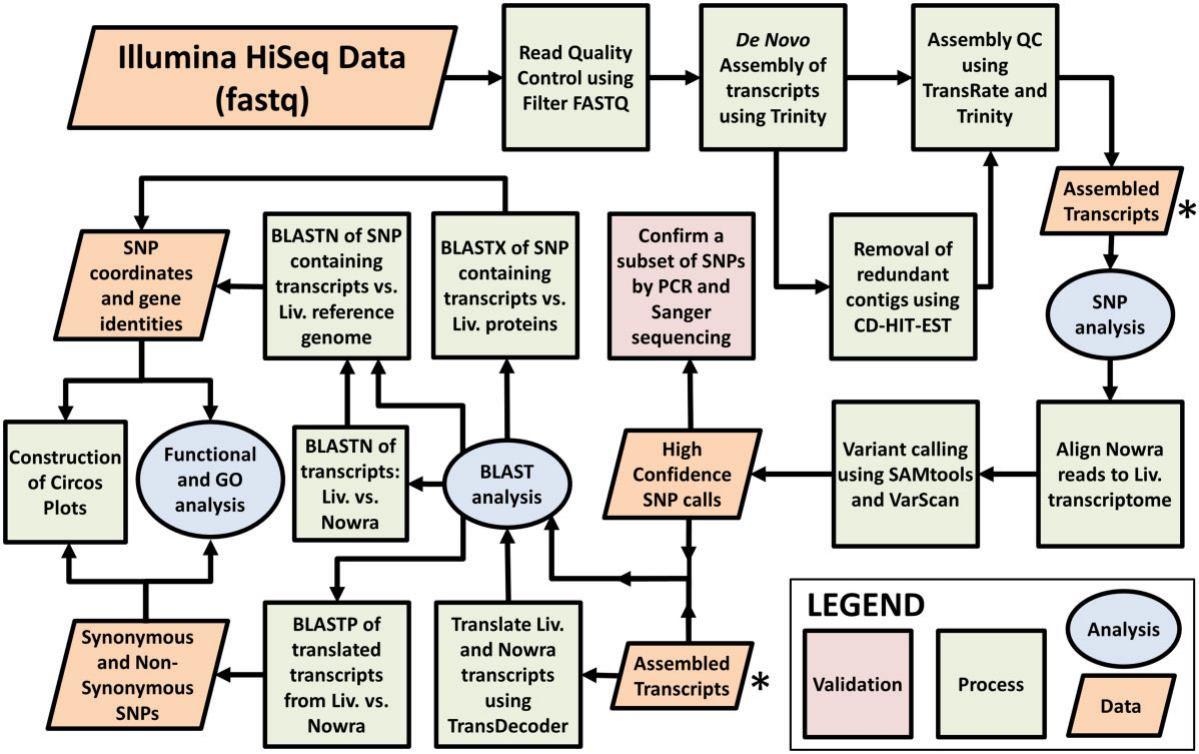
—A summary of the complete variant detection workflow used in this study. Beginning with NGS (Illumina HiSeq) data, the reads generated from NC-Liverpool and NC-Nowra were used to assemble individual transcriptomes, and identify sequence variations between the two strains. A subset of high confidence variants was subsequently selected for laboratory validation for a total of ten *N. caninum* strains, each of which differs in pathogenic capability, geographical origin, and source. The SNP callset was then subjected to various computational analyses to determine their genomic location and functional significance, identify highly polymorphic regions, reveal whether their presence resulted in nonsynonymous (missense) or synonymous (silent) mutations, and determine an underlying population structure.

A summary of the workflow discussed above in its entirety is presented in figure 1, including the data sets exploited and created, the tools and software employed, and the analyses conducted.

### Population Structure

To investigate whether an underlying population structure existed amongst the *N. caninum* strains studied based on identified sequence polymorphisms, a neighbor-joining tree was generated from a genetic distance matrix using the neighbor-joining tree estimation method of Saitou and Nei (1987). This was performed with the “nj” function within the “ape” R package, using the sequencing data generated through PCR analysis for each isolate as input.

DNA extracted from cultured *N. hughesi* tachyzoites was subjected to PCR amplification and sequencing of the same polymorphic loci, to investigate whether the confirmed variants identified in this study for *N. caninum* isolates were present. The sequences were used to generate an additional neighbor-joining tree incorporating the ten *N. caninum* strains, as well as *N. hughesi*.

### Comparison to *Toxoplasma gondii* Markers

Genetic markers commonly used in RFLP analyses of *T. gondii* were identified and cross referenced to the *N. caninum* SNP callset, in an attempt to elucidate whether the two closely related species were comparable with respect to genotyping markers (Lorenzi et al. 2016; Ruffolo et al. 2016). Additionally, based on the current understanding of genomic variation exhibited among the four major *T. gondii* lineages (Boyle et al. 2006; Khan et al. 2006; Khan et al. 2011b), the genes located on chromosome Ia in *N. caninum* were examined to see whether SNPs identified in this study mapped to this locus.

## Results

### Generation of a Reference Transcriptome

VarScan identified 1,520 high confidence SNPs following mapping of the NC-Liverpool transcriptome reads to the published NC-Liverpool reference genome. A total of 12 SNPs from this callset were subsequently confirmed through PCR analysis and Sanger sequencing as true differences between the two sources (see supplementary file S1, table S3, Supplementary Material online). The NC-Liverpool DNA sequenced from a cryopreserved 1998 NC-Liverpool culture (sourced from Liverpool University [Barber et al. 1993]) and the 2017 NC-Liverpool passage sequenced for this study were identical at these SNP locations. This confirmed the genetic stability of the strain over time, and indicated that the NC-Liverpool strain cultivated in-house was either genetically distinct from the published NC-Liverpool genome, or that the published NC-Liverpool genome contained some erroneous SNPs. This led us to use our in-house de novo transcriptome assembly as our reference for variant calling, given we could validate it by Sanger sequencing. Following removal of redundant contigs using CD-HIT-EST, 45,297 transcripts (27,570,740 assembled bases) remained in the Trinity assembly for use as a reference for mapping NC-Nowra reads and subsequent variant calling. A summary of the assembly metrics is contained within supplementary file S1 and S2, Supplementary Material online.

### Variant Calling

The NC-Nowra and the NC-Liverpool tachyzoite transcriptomes differed by 3,130 SNPs and 6,123 indels (table 1). Supplementary file S3, Supplementary Material online, contains a list of NC-Liverpool transcriptome contigs containing SNPs identified by VarScan, along with the variant positions, and reference and alternate bases. The number of SNPs observed in a contig ranged from 0 to 28, the average being 1.55 SNPs per contig. A total of 1,838 transitions (A/G and C/T) and 1,292 transversions (A/C, A/T, C/G, and G/T) were observed between the NC-Nowra and NC-Liverpool strains, representing a transition/transversion ratio (T_i_/T_v_) of 1.42. The 3,130 high confidence SNPs were distributed across 2,021 unique transcripts encoded by 1,879 genes. Additionally, the current *N. caninum* reference genome consists of multiple large contigs that are not assigned to one of the 14 chromosomes. There were 162 SNPs distributed across 22 such contigs, with the majority of this callset (∼78%) assigned to eight of these contigs alone. These SNPs were subsequently allocated to 34 unique protein-coding genes. It is worth noting that when blasting the NC-Liverpool transcriptome against the published *N. caninum* annotated proteins to assign SNPs to annotated genes, several contigs containing SNPs returned high confidence BLAST hits (i.e., PID ≥ 90%) to multiple genes along the genome, however not all SNPs were assigned to protein-coding genes based on the BLAST results.

**Table 1:** Summary of VarScan Variant Calling Using NC-Nowra RNA-seq Reads Aligned to the *De Novo* NC-Liverpool Transcriptome Reference

	Number of SNPs Called		Number of Indels Called	
	Prefiltering	Postfiltering	Prefiltering	Postfiltering
Pre-CD-HIT-EST	15,807	3,562	8,163	5,067
Post-CD-HIT-EST	15,361	3,130	8,966	6,123

Note.—Most SNPs initially called by VarScan were discarded following filtering based on strand bias, sequence, and variant coverage thresholds, mismatch quality sum, and read position bias.

A set of 27 variants identified in silico between NC-Liverpool and NC-Nowra were subject to PCR and sequencing analysis (table 2). No false-positive variants were identified from among the 27 variants examined, though the workflow failed to detect three true variants (i.e., three false negative SNPs) within these genetic markers, as revealed by Sanger sequencing. It was found that VarScan originally identified these variants, but they were discarded during subsequent filtering steps.

**Table 2:** A Summary of the Total Number of Variants Selected for and Confirmed Through Sequencing, Based on Targeting Various Loci

Metric	Value
High confidence SNPs called by VarScan between NC-Liverpool transcriptome and NC-Nowra reads	3,130
Variants captured and confirmed through Sanger sequencing	37
Variants captured and confirmed in MLST targets	27
Variants captured in MLST, identified as false positives through Sanger sequencing	0
Variants discovered via Sanger sequencing, identified as false negatives by VarScan	3
Total variants captured in MLST, sequenced for a total of ten *N. caninum* strains	30

### Distribution and Functional Annotation of SNPs

**Fig. 2.**
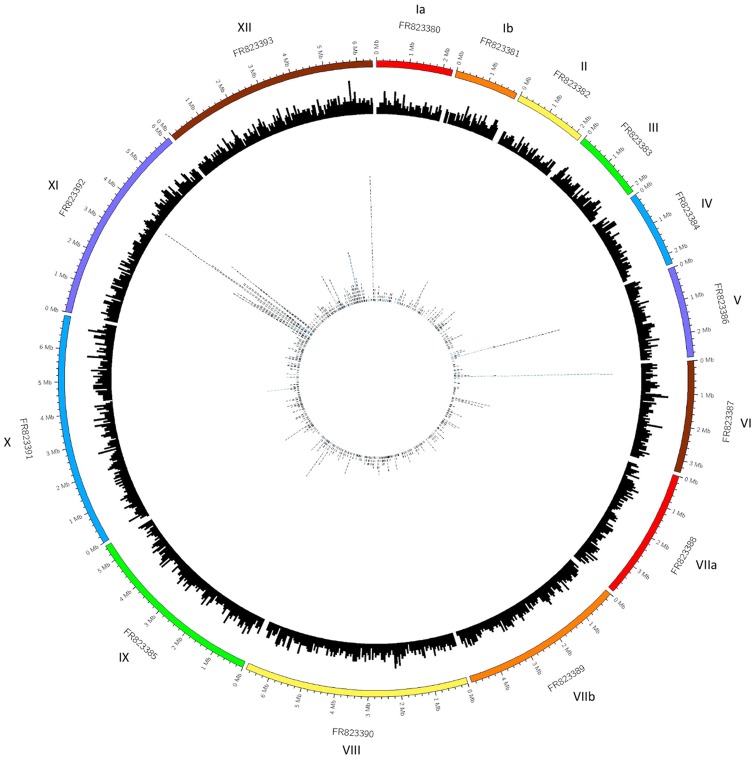
—Circos plot representing the SNP data in the context of the *N. caninum* genome. The outer track is an ideogram representing the 14 *N. caninum* chromosomes and their sizes, followed by a histogram of the 7,121 annotated genes along each chromosome in the middle track. This histogram is based on each gene’s location within a chromosome, plotted in 50 kb windows, relative to the ideogram. The inner most track contains the distribution of identified SNPs as located within these annotated genes. Each tile in this inner track represents a SNP that has fallen within that gene region, relative to the ideogram. The tiles are also colored based on their size, where those that are blue represent genes that are larger than 10,000 bases.

Multiple SNP hotspots were identified, distributed unevenly throughout the *N. caninum* genome (fig. 2). A large number of SNPs clustered on chromosome XI (FR823392), in addition to various hotspots identified in chromosomes V (FR923386), VI (FR823387), and XII (FR823393). There were 19 hotspots containing 73 *N. caninum* genes, many of which were implicated in translation (*NCLIV_057380* and *NCLIV_057360*), transcription (*NCLIV_057870* and *NCLIV_065940*), ribosomal subunit formation (*NCLIV_056680*, *NCLIV_056820*, *NCLIV_056830*, and *NCLIV_057070*), GTP binding and GTPase activity (*NCLIV_057820* and *NCLIV_057390*), protein transport (*NCLIV_057490*), and kinase activity or protein phosphorylation (*NCLIV_ 056620* and *NCLIV_057940*). The genomic location of these 73 genes contained within SNP hotspots and their annotations are tabulated in supplementary file S1, table S5, Supplementary Material online. Thirty-five genes from this callset contained five or more SNPs.

Prior to functional analysis, 23 of the 35 SNP hotspot genes were identified as hypothetical proteins or unspecified products based on their corresponding gene IDs. All but nine were assigned GO terms, protein families, domains and/or repeats by InterProScan. Protein superfamilies that appeared more than once among these 35 SNP hotspot genes included WD40 repeat containing domain superfamily (IPR036322), ARM-like helical (IPR011989), ARM-type fold (IPR016024), and P-loop-containing nucleoside triphosphate hydrolase (IPR027417). Other superfamilies of functional interest were zinc finger RING/FYVE/PHD type (IPR013083), Sec1-like superfamily (IPR036045), EF-hand domain pair (IPR011992), ABC transporter superfamily (IPR036640), and the translation initiation factor eIF-4e-like (IPR023398) superfamilies. Domains and repeats featured were AAA+ ATPase domain (IPR003593), tetratricopeptide repeat (IPR019734), subtilisin SUB1-like catalytic domain (IPR034204), and WD40-repeat-containing protein (IPR017986).

Recurring Gene Ontologies (GO) for molecular function included protein binding (GO: 0005515), binding (GO: 0005488), and hydrolase activity (GO: 0016787). Regarding biological process GOs, those assigned included lipid metabolic process (GO: 0006629), translation initiation (GO: 0006413), metabolic process (GO: 0008152), proteolysis (GO: 0006508), and transmembrane transport (GO: 0055085). Supplementary table S6 within supplementary file S1, Supplementary Material online contains a complete list of gene annotation information and ontologies for the putative proteins encoded within these SNP hotspots.

The three bioinformatic tools employed to identify transmembrane (TM) proteins and signal peptides within the SNP hotspot list, did not present consistent results for all protein sequences explored. However, mutually reported between both Phobius and Philius, were four transmembrane proteins, and six globular proteins with signal peptides, all of which were present on either chromosome VI or XI, except for one signal peptide containing protein which was located on chromosome V. Two hotspot genes also encoded transmembrane proteins with signal peptides, both of which were located on chromosome XI (*NCLIV_056900* and *NCLIV_057550*). Interestingly, two TM proteins and two signal peptide containing proteins could not be assigned any additional annotations or gene ontologies.

### Estimation of Synonymous and Nonsynonymous SNP Count

**Fig. 3.**
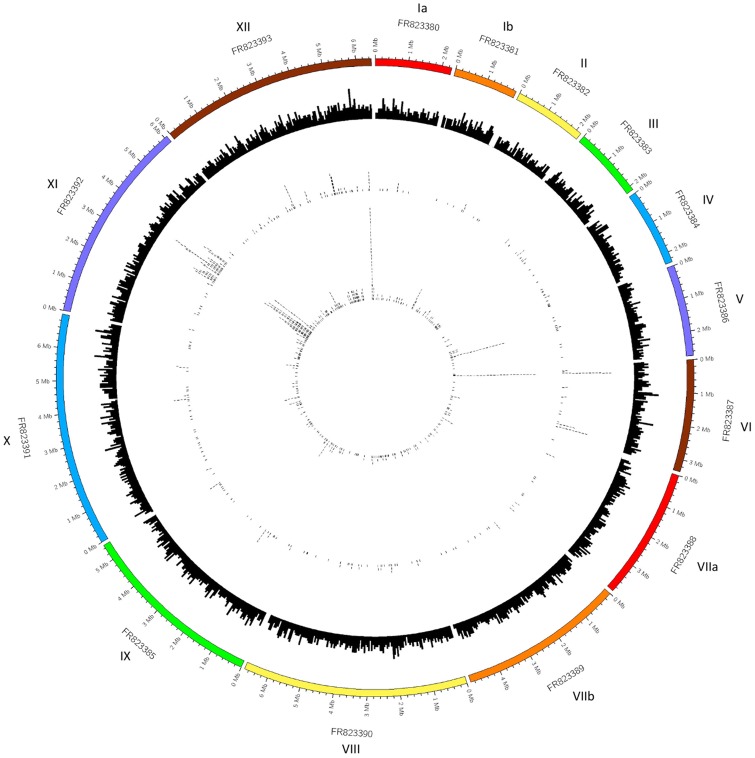
—Circos plot presenting the location of nonsynonymous SNPs identified in the *N. caninum* genome. The outer track is an ideogram, representing the 14 *N. caninum* chromosomes and their sizes, followed by a histogram of the 7,121 annotated genes along each chromosome on the second track from the outside. This histogram is based on each gene’s location within a chromosome, plotted in 50 kb windows, relative to the ideogram. The next track (third from the outside) represents the locations of nonsynonymous SNPs called by VarScan within these annotated genes. Similarly, the innermost track depicts the locations of synonymous SNPs identified by VarScan.

When the translated transcriptomes of NC-Nowra and NC-Liverpool were compared, 652 SNPs were found to be located in open reading frames that possessed different translations between the strains, and these SNPs were distributed across 287 unique genes. There were also 470 SNPs assigned to a protein-coding gene where the translation of the respective transcript was identical between the strains (i.e., synonymous mutations). However, where the number of mismatches reported by BLASTP exceeded the number of SNPs within a contig, it was assumed that VarScan had filtered out real sequence variants between the two strains (i.e., false negatives SNPs). Alternatively, in the event that there were more SNPs identified by VarScan within a contig than BLASTP mismatches, the additional SNPs were presumed to result in synonymous mutations. Therefore, it was estimated that the final VarScan SNP callset contained at least 468 nonsynonymous SNPs, and 654 synonymous SNPs. Figure 3 displays the distribution of the nonsynonymous and synonymous SNPs identified across the *N. caninum* genome. Many nonsynonymous mutations coincided with the locations of the SNP hotspots identified, including those on chromosomes VI (FR823387), XI (FR823392), and XII (FR823393), whereas almost all the SNPs located on chromosome V (FR823386) were found to be synonymous mutations. Additionally, the aforementioned callsets included 60 nonsynonymous SNPs, and 63 synonymous SNPs part of large contigs within the *N. caninum* genome, which are not pictured in the Circos plots generated.

### Functional Analysis of Transcripts Containing nonsynonymous SNPs

The GOs that were overrepresented in transcripts containing nonsynonymous SNPs from amongst the molecular function GO category included protein kinase activity (GO: 0004672), ATP binding (GO: 0005524), and protein binding (GO: 0005515). Recurring GOs from the biological process category included protein phosphorylation (GO: 0006468), proteolysis (GO: 0005576), and oxidation–reduction process (GO: 0055114). The protein superfamilies repeatedly featured were protein kinase-like domain superfamily (IPR011009), p-loop containing domain-like superfamily (IPR027417), WD40-repeat containing domain superfamily (IPR036322), and tetratricopeptide-like helical domain superfamily (IPR011990). Recurring protein domains of functional importance included protein kinase (IPR000719), AAA+ ATPase (IPR003593), EF-hand calcium binding (IPR018247), and PAN/Apple domain (IPR003609), as well as featured protein repeats such as WD40 repeat (IPR001680), and Ankyrin repeat (IPR002110). Also of interest as reported by InterProScan, were protein signatures such as serine/threonine protein kinase active-site signature, protein kinase ATP binding site signature, protozoan surface antigen signature (SAG1), and ABC transporters family signature.

Twenty-seven of the 35 SNP hotspot genes were found to contain nonsynonymous SNPs, including proteins coding for kinesin, SUB2, an ABC transporter, a Sec1 protein, and fatty acyl-CoA desaturase.

### Distribution of Transcripts of High and Low SNP Densities

**Fig. 4.**
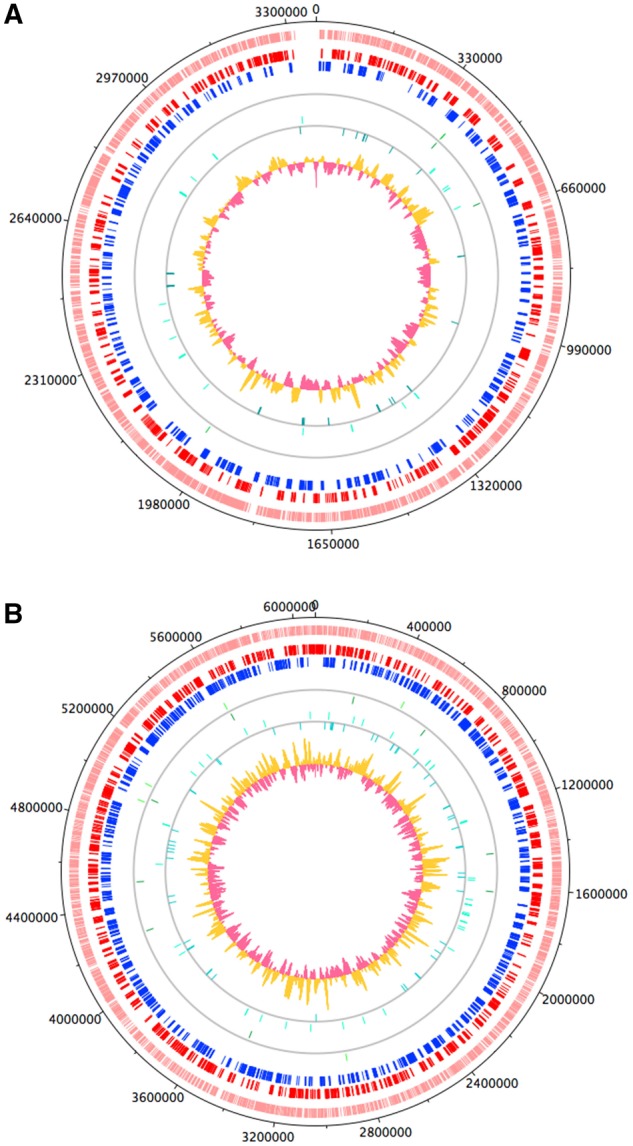
—Plots displaying the features of chromosome VI (*A*) and XI (*B*). The outer most track (pink) in both plots represents the location of all genes on either chromosome. The adjacent two tracks contain the CDS on the forward (red) and reverse (blue) strands. The green strokes on the next two tracks show the location of tRNAs on the forward and reverse strands, respectively, and similarly the location of ncRNAs are represented by the aqua strokes on either strand. The GC content along the chromosome is displayed in the second most inner track, where the yellow depicts areas above average content, and the pink being below average.

Chromosome XI (FR823392) possessed the largest number of contigs with a high SNP density across the genome. The *z*-test confirmed that the two chromosomes encoding a significantly larger number of SNP-dense transcripts (*P* value <0.05), compared with the number of contigs with a low SNP density, were chromosomes VI (FR823387) and XI (FR823392). Figure 4*A* and *B* depict the main genomic features of chromosomes VI and XI, plotted using the available GenBank records for chromosome VI (LN714480.1) and XI (LN714480.1). Both chromosomes are transcriptionally active in *N. caninum* tachyzoites, and only a very small number of noncoding regions exist between genes. The SNP hotspots within these chromosomes seem localized to selected genomic windows. The chromosomes also encode ncRNA (noncoding RNA) molecules, including tRNAs, dispersed unevenly along the length of each chromosome. Additionally, there are clear areas where the GC content along the chromosome either peaks above average, or decreases. Some SNP hotspots on either chromosome also appear to coincide with peaks in GC content, such as that on chromosome VI between approximately 450,000 and 500,000 bases.

### Genetic Population Structure

**Fig. 5.**
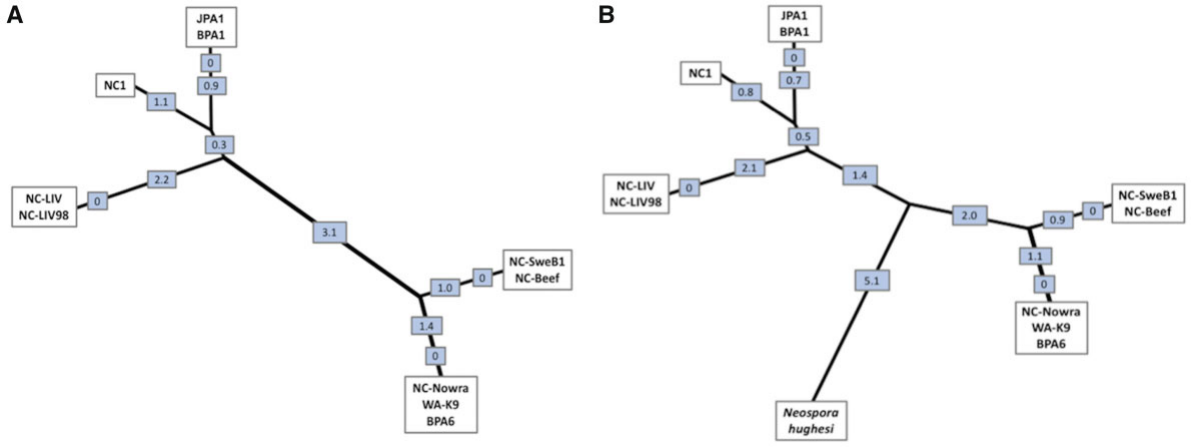
—Unrooted neighbor-joining trees showing the population structure within the *Neospora* genus. (*A*) This unrooted neighbor-joining tree was generated from pairwise genetic distances calculated from nine genetic markers capturing 30 variants, including ten *N. caninum* strains (including one repeat of NC-Liverpool; NC-LIV98). The tree suggests a population genetic structure comprising two major lineages of *N. caninum*. (*B*) This neighbor-joining tree was generated from seven of the nine genetic markers capturing 28 confirmed variants for *N. caninum* strains, as well as an additional 28 SNPs that were unique only within the *N. hughesi* sequences. The values displayed in both trees indicate the genetic distance between nodes.

The Sanger data generated for eight additional *N. caninum* strains, across nine selected loci (NC1, JPA1, NC-SweB1, WA-K9, NC-Beef, BPA1, BPA6, and an additional NC-Liverpool strain cryogenically frozen since 1998) containing 30 of the confirmed variants, did not reveal any specific patterns of segregation (i.e., geographical or otherwise). Nevertheless, JPA1, BPA1, and NC1 were more similar to NC-Liverpool, whereas NC-Nowra, NC-SweB1, NC-Beef, BPA6, and WA-K9 were more similar to each other than to the formerly mentioned isolates. The NC-Liverpool strains from different passage numbers were identical. The neighbor-joining tree presented in figure 5*A* revealed the grouping of the ten strains into two distinct clades based on the SNP data: the virulent strains including NC-Liverpool, and the more attenuated group including NC-Nowra and NC-SweB1.

Seven of the nine sequenced polymorphic loci (supplementary file S1, table S4, entries 1–7, Supplementary Material online) which contained 28 confirmed variants between NC-Liverpool and NC-Nowra identified in this study, were able to be amplified for *N. hughesi*, through the DNA extraction and PCR amplification methodology described. The sequencing results for *N. hughesi* revealed the presence of all 28 variants identified in silico for *N. caninum*, as well as the existence of an additional 28 SNPs that were unique to *N. hughesi*. Presented in figure 5*B* is an additional neighbor-joining tree generated from seven of the nine aligned polymorphic loci, for *N. hughesi* and all ten *N. caninum* strains. These results provide further additional support for the existence of two clades of *N. caninum*.

### Comparison to *Toxoplasma gondii* Markers

Of the 12 *T. gondii* genotyping markers examined, only two SNPs were present within *N. caninum* orthologous genes. They were in dense granule protein (*GRA7*) and class I chitinase (*CLP1*). These results appear to be consistent with that of Al-Qassab et al. (2010b), where no sequence differences were detected in various proteins of canine and bovine *N. caninum* strains. This included *SAG1*, *SRS2*, and *GRA6*, all of which are among the 12 *T. gondii* genotyping markers explored in this study. Due to the location of one SNP at the very beginning of the contig, PCR and sequencing analysis was only performed and confirmed for one of these SNPs, as per the last entry in supplementary table S4 of supplementary file S1, Supplementary Material online.

After assigning the location of each SNP to a chromosome (fig. 2) to examine the distribution of variation along the *N. caninum* genome, it was observed that chromosome Ia (FR823380) had the second lowest SNP density with <100 SNPs being present, only second to chromosome Ib (FR823381).

## Discussion

*Neospora caninum* is an apicomplexan parasite, responsible for reproductive failure in cattle and neurological disease in dogs. Intraspecies diversity is known in the form of extreme differences in virulence between strains found across the globe. The genetic basis of this diversity is unknown though an improved understanding of this could help to identify novel virulence loci.

We used a bioinformatics workflow to identify genome-wide genetic differences between two phenotypically distinct strains of *N. caninum*. These strains vary drastically in their pathogenic propensity, and represent extremes of *N. caninum* virulence (Atkinson et al. 1999; Miller et al. 2002). A variant analysis workflow was employed to identify SNPs present in the genomes of NC-Liverpool and NC-Nowra, and the SNPs were subsequently subjected to laboratory validation through PCR and Sanger sequencing. A multilocus sequencing approach was developed using this information, comprising of nine randomly selected loci, with a combined length of 3.4 kb and containing 30 validated variants. This method was applied to ten *N. caninum* strains, including two NC-Liverpool samples from different passages and NC-Nowra, to reveal a population structure consisting of two major clades. We also identified SNP hotspots within the genome of *N. caninum*, characterized by elevated levels of SNP density.

The choice of variant caller for genome-wide SNP detection requires careful consideration and optimization depending on the organism under investigation and the data available (Reumers et al. 2012; O'Rawe et al. 2013; Bao et al. 2014; Pabinger et al. 2014; Ribeiro et al. 2015). In addition, some variant callers such as the routinely used Genome Analysis Toolkit (GATK) (McKenna et al. 2010) require a database of known SNPs to preprocess reads for variant calling, and consequently fail to consider nonmodel organisms such as *N. caninum*. As the majority of variant callers are like the GATK and depend on reference-based mapping, their use in nonmodel species is often restricted due to the absence of high-quality reference genomes (Dou et al. 2012).

The VarScan package employed in this study exploits empirical and statistical thresholds based on user-defined criteria to call variants, representing a simple pipeline that is compatible with several short-read aligners (Koboldt et al. 2013). This versatility means it can be applied to nonmodel organisms such as *N. caninum*. Using its default recommended parameters, the VarScan 2 pipeline (Koboldt et al. 2013) identified thousands of high confidence SNPs and indels between the de novo NC-Liverpool transcriptome generated in-house, and the NC-Nowra RNA-seq data. Of the hundreds of variants that were randomly selected for manual visualization in IGV, most exhibited robust quality scores and high sequence coverage at the variant position. Sanger sequencing identified a small number of false negative variants filtered out subsequent to variant calling. This highlights the need for careful optimization of filtering parameters and the necessity of validating SNPs identified *in silico* by Sanger sequencing before deriving any biological conclusions.

Ribeiro et al. (2015) explored the relationship between the choice of tools and parameters, and their impact on false positive variants. Out of the seven factors explored, the quality of the reference sequence used had the most pronounced effect on the false positive variant calling rate. This finding raises concerns for the use of similar variant calling pipelines on nonmodel organisms in the early stages of genomic examination where the reference genomes may be poor or misassembled, the product of limited or incomplete sequencing, or the result of inadequate quality control and validation. This can subsequently result in errors in the reference sequence being identified as read mismatches, producing false positive variants.

Using the NC-Liverpool genome from ToxoDB as a reference, all the variants called were false positives, typically located at the ends of reads or in homopolymer runs, which are known error sources associated with DNA sequencing (Reumers et al. 2012; Durtschi et al. 2013). This discovery prompted the assembly of a de novo transcriptome using RNA-seq data derived from the NC-Liverpool parasites cultured in-house. The variant calling workflow employed here identified numerous SNPs when comparing our cultured NC-Liverpool strain to the ToxoDB NC-Liverpool reference, suggesting the reference in ToxoDB was erroneous. Although alternatively it is possible that such identified variants could represent allelic variation between these NC-Liverpool cultures, this does not seem to be the case, at least for the set of 12 variants that were confirmed through sequencing. Based on our extensive work and the original use of the ToxoDB NC-Liverpool genome as a reference for variant calling, the confirmed SNPs are most likely attribulable to errors in this published genome. The absence of any differences between the 2017 and 1998 NC-Liverpool strains based on our MLST approach also supports this.

Analysis of the distribution of identified SNPs elucidated the existence of SNP hotspots across the *N. caninum* genome (fig. 2), especially their clustering on chromosome VI (FR923387), XI (FR923392), and XII (FR823393). However, the current (or absence of) annotation of the *N. caninum* genome presented a problem for assigning functional significance to the SNPs identified in this study, and more broadly remains a problem for the study of virulence and pathogenicity within the species. The fact that 4,011 of 6,936 genes in the published *N. caninum* genome are annotated as hypothetical proteins, presents a major and concerning hindrance to the study of potential virulence factors. Furthermore, recent studies focusing on improving and expanding the available gene structure and annotations for *N. caninum* are yet to appear in ToxoDB reference resources (Goodswen et al. 2015; Krishna et al. 2015; Ramaprasad et al. 2015). Although 3,130 high confidence SNPs were called and 19 genomic SNP hotspots identified, many were located within the coding regions of hypothetical proteins or uncharacterized genomic regions, which greatly hindered the ability to assign biological context to these polymorphic regions.

In an effort to annotate the corresponding protein sequences for each SNP hotspot identified in this study, many of which were hypothetical proteins, various tools such as InterProScan were used. Within these hotspots were two genes coding for WD40 domain containing protein: *NCLIV_057900* and *NCLIV_013170*. WD40 repeat containing proteins belong to one of the largest, most abundant protein families found in all eukaryotes (Neer et al. 1994). These proteins are associated with a variety of functions including signal transduction and transcription regulation, cell cycle control, autophagy, apoptosis, transmembrane signaling, and cytoskeleton assembly. The fundamental shared function of all WD40-repeat proteins is facilitating multi-protein complexes, where the repeats serve as a rigid scaffold for protein interactions. The significance of this is that for intracellular protozoan parasites, the efficiency of infection is contingent on the parasite’s capacity for host cell recognition, adhesion, and invasion, which are generally mediated by protein–protein interactions (von Bohl et al. 2015).

InterProScan characterized one hypothetical, SNP hotspot protein (*NCLIV_057320*) as belonging to the tetratricopeptide-like helical domain superfamily. As with members of the WD40 family, TPR containing proteins are involved in protein–protein interactions and various metabolic and regulatory processes, and thus play an important role in virulence (Goebl and Yanagida 1991). Also amongst the most abundant proteins in eukaryotes, and characterizing one identified hotspot gene, zinc finger domain containing proteins exhibit versatile binding modes, suggesting that such motifs are stable scaffolds with specialized functions. Zinc finger proteins are involved in transcription and translation regulation, DNA and RNA recognition, protein folding and assembly, apoptosis, and cell adhesion (Laity et al. 2001).

The ATP binding cassette (ABC) superfamily of proteins are expressed as efflux transporters in eukaryotes, that translocate a plethora of substrates including proteins, ions, toxins and amino acids across membranes (El-Awady et al. 2017). All ABC transporters consist of two domains: the nucleotide binding domain (NBD) and the (transmembrane domain [TMD], where the coupling of these domains facilitates import and export). The protein hotspot identified as an ABC transporter, *NCLIV_065950*, had gene ontologies related to transmembrane transport (GO: 0055085), ATP binding (GO: 0005524), and ATPase activity coupled to transmembrane movement of substances (GO: 0042626). However, while Phobius recognized the transmembrane topology of this protein coding sequence, Philius and TMHMM did not.

Since the data exploited in this study was generated from RNA-seq data, it was unexpected that SNPs were identified that were not located in annotated genes. This suggests that either the current gene annotation is incorrect or incomplete, or that new/novel abundantly expressed transcripts were present in the culture from which the RNA-seq data was generated. However, this study did not investigate the presence of sequence variants located within apicoplast or mitochondrial DNA, to which some of the identified SNPs may have been located within.

In addition to the mutational hotspots revealed throughout the *N. caninum* genome in this study, the nonsynonymous mutations identified can also contribute to the current understanding of pathogenic variability within the species. As a nonsynonymous SNP alters a protein’s sequence, their presence can cause changes in biochemical activity, protein–protein interactions, and molecular function, which can consequently establish the link between genotype and biologically significant phenotypes (Ng and Henikoff 2006; Zhao et al. 2014; Tang and Thomas 2016). This stresses the importance of not only identifying and comparing sequence variants present between populations, but also understanding whether such mutations have the potential to disrupt the resulting protein’s function. The identification of nonsynonymous SNPs within protein coding genes in this study may provide new insight into and sources for studying the underlying causes of phenotypic differences between isolates of *N. caninum*, presenting new potential determinants of virulence and pathogenic capability.

Analyzing and recognizing the existence of population structure within a species is conducive to understanding and determining the spread of virulence factors within and between geographic locations (Khan et al. 2011b). As presented in figure 5*A*, the ten strains, including two NC-Liverpool strains from different passages, comprise two distinct genetic clusters that may reflect differences in pathogenicity. The highly virulent NC-Liverpool strain was the most distinct type, and was placed at a genetic distance furthest from the clades containing the less virulent NC-Nowra and NC-SweB1 strains, but at a small distance from the virulent NC1 strain. Although significant differences in virulence between select *N. caninum* strains have been published in either mice or cattle models (Dubey et al. 1992; Atkinson et al. 1999; Innes et al. 2001; Miller et al. 2002; Maley et al. 2003), limited studies currently exist that comprehensively document the pathogenic variability of many other isolated strains, including NC-Beef, BPA6, and WA-K9. This makes it difficult to corroborate the population structure elucidated in this study and make an assumption regarding virulence, based on the presence or absence of sequence variations investigated. However, the neighbor-joining tree presented in figure 5*B* with the inclusion of *N. hughesi*, supports the existence of a two-clade population structure for *N. caninum*, dividing the ten strains into genetic clusters potentially resembling their virulence properties. We refrain from suggesting that *N. caninum* as we know it, may represent two independent species. The relationship represented in figure 5*B* including *N. hughesi* suggests that this idea is worth investigating further. It is also worth mentioning that the two clades elucidated in this study reflect the results of the Tand-12 minisatellite marker described by Al-Qassab et al. (2010a) for these isolates. The NC-Liverpool cluster contained three copies of this repeat, whereas the NC-Nowra cluster is characterized by four copies of this repeat.

Fatality was observed in only one of eight susceptible γ-INF-KO mice infected with NC-Beef oocysts, Lindsay et al. (1999) suggested that this strain may be characterized by a lack of pathogenicity. Additionally, WA-K9 was the first canine strain from Australia, cultivated from skin lesions found on a dog in Western Australia (McInnes et al. 2006). What was noteworthy about the clinical presentation of this dog was that infection initially manifested as cutaneous neosporosis, where the parasite is primarily responsible for neurological illness in canines. However, the dog was essentially normal at a 2.5 year follow up examination after continuous treatment with a high dosage of clindamycin, and subsequent to initial treatment and recrudescent infection. The successful treatment and opportunistic infection characterizing this particular case, may suggest reduced virulence of this strain, and hence further affirm the population structure determined in this study.

The NC-Liverpool DNA sequenced from the 1998 culture (sourced from Liverpool University [Barber et al. 1993]) and the 2017 passage sequenced for this study were identical at the genomic locations studied in MLST, confirming the genetic stability of the strain over time, and indicating that the NC-Liverpool cultivated in-house was either genetically distinct from the published NC-Liverpool genome, or that this genome contains erroneous SNPs. It is also important to note that the 1998 isolate is known to be virulent in mice (Atkinson et al. 1999). Additionally, the absence of virulence in the NC-Nowra isolate was confirmed as recently as 2013 in cattle vaccine trials (Weber et al. 2013). However, it should be noted that this study did not compare other isolates previously categorized as virulent in cattle, such as NC-Liverpool.

Although studies have established varying degrees of intraspecies genetic diversity within *N. caninum* in repeat regions, it is expected that SNPs will replace repetitive sequences as DNA markers, due to their distribution throughout the entire genome and their low mutation rates (Picoult-Newberg et al. 1999). As variant identification using RNA-seq data from *N. caninum* is unprecedented, it is valuable to compare these results to genetic variation identified in well-studied model organisms. *Toxoplasma gondii* is a model Apicomplexan with robust data available, and has been thoroughly studied to elucidate existing genotypes, population structure, and potential virulence markers. The plethora of studies exploring the population structure of *T. gondii* has shown that the global between-lineage variation ranges from approximately 0.01 to 5% (Boyle et al. 2006). It is well documented that a distinct split exists between *T. gondii* lineages found in North America and Europe, compared with those in South America (Khan et al. 2011b). Furthermore, the cause of most infections in the Northern Hemisphere can be traced to four clonal lineages, each with differing levels of pathogenicity (Khan et al. 2011a). It was observed that very few sequence polymorphisms exist on chromosome 1a between these dominant lineages (Khan et al. 2006, 2011b). Due to this common monomorphic chromosome, the current model of evolution suggests that approximately 10,000 years ago a genetic sweep caused the expansion of these lineages, from only a limited number of genetic crosses between highly related precursor strains (Boyle et al. 2006). After assigning the location of each SNP to a chromosome (fig. 2), it was observed that chromosome Ia in *N. caninum* had the second lowest SNP density with <100 SNPs being present. Whether *N. caninum* experienced a similar genetic sweep to *T. gondii* at the time is not entirely clear, however if this was the case, based on the existence of SNPs across this locus, such a sweep may not have been as severe.

In summary, this study shows that variant analysis can contribute to our understanding of the existence and underlying mechanisms of genetic diversity within the *N. caninum* species, as well as the mechanisms of virulence and pathogenesis. Based on this, SNP identification has the potential to replace mini- and microsatellite markers for exploring such intraspecies diversity. The MLST approach developed in this study reveals a population structure reflecting two major clades that do not support any obvious geographical segregation. This knowledge will facilitate the future identification of novel virulence markers and guide the selection of candidate components for a subunit vaccine against bovine neosporosis.

In addition, we present a bioinformatic workflow that identified thousands of genetic variants in loci that are transcriptionally active during the tachyzoite stage of the *N. caninum* life cycle. This data informed the development of an MLST approach based on nine transcriptionally active tachyzoite-associated loci that provides new insights on the population genetic structure of *N. caninum*. We also identify a set of *N. caninum* proteins as potentially novel virulence determinants for downstream investigation, based on both the presence of SNP-dense regions (hotspots), and nonsynonymous mutations within protein-coding genes. This work provides new insights into the molecular basis behind the marked virulence properties reported between strains of *N. caninum*, which is knowledge that will be pertinent to the future development of a subunit vaccine against bovine neosporosis.

## Acknowledgments

We would like to thank Martin Krzywinski for his assistance with the Circos software. This study was completed by L.C. in partial fulfilment of the Ph.D. degree at UTS.

## Supplementary Material

Supplementary Data
