# Supplementary material for: Genome Wide Identification of Mutational Hotspots in the Apicomplexan Parasite Neospora caninum and the Implications for Virulence
Source: Genome Biol Evol. 2018 Aug 25;10(9):2417–31. doi: 10.1093/gbe/evy188 (PMC6147731; doi:10.1093/gbe/evy188)
Supplement: Supplementary Data [file evy188_supp.zip › evy188_Supp/Supplementary File 1 - Methods & Results.docx]

This file provides the supplementary materials accompanying the following manuscript:

**Genome Wide Identification of Mutational Hotspots in the Apicomplexan Parasite *Neospora caninum* and the Implications for Virulence**

Larissa Calarco^1*^, Joel Barratt^1^, John Ellis^1^

^1^ University of Technology Sydney, School of Life Sciences

Table of Contents

[Supplementary Methods 2](#_Toc514842627)

[Ensuring paired information was retained following read QC 2](#_Toc514842628)

[Optimisation of TopHat alignment parameters 3](#_Toc514842629)

[Variant Calling 3](#_Toc514842630)

[De novo transcriptome assembly 5](#_Toc514842631)

[Building a BLAST database to annotate variant positions 6](#_Toc514842632)

[Supplementary Results 7](#_Toc514842633)

[De novo transcriptome assembly with Trinity using NC-Liverpool RNA-seq data 7](#_Toc514842634)

[Primer Design 8](#_Toc514842635)

[Annotation of SNP hotspots regions 10](#_Toc514842636)

# Supplementary Methods

**Table S1. List of *N. caninum* isolates used in this study.**

| ***N. caninum* isolates** | **Country** | **Source** | **Reference** |
| --- | --- | --- | --- |
| NC-Liverpool | United Kingdom | Cerebrum of dog | (Barber, et al. 1993; Barber, et al. 1995) |
| NC-Nowra | Australia | Brain and spinal cord of calf | (Miller, et al. 2002) |
| NC1 | USA | Brain of dog | (Dubey, et al. 1988) |
| JPA1 | Japan | Brain and spinal cord of calf | (Yamane, et al. 1996; Yamane, et al. 1997) |
| NC-SweB1 | Sweden | Brain of stillborn calf | (Stenlund, et al. 1997) |
| WA-K9 | Australia | Skin lesions of dog | (McInnes, et al. 2006) |
| NC-Beef | USA | Calf | (McAllister, et al. 1998) |
| BPA1 | USA | Brain of aborted calf | (Conrad, et al. 1993) |
| BPA6 | USA | Brain and spinal cord of aborted calf | (Conrad, et al. 2004) |

##

## Ensuring paired information was retained following read QC

The following Perl script, available from GitHub (https://github.com/broadinstitute/viral-ngs/blob/master/tools/scripts/mergeShuffledFastqSeqs.pl.), was run to ensure paired read information was preserved following read grooming and quality trimming:

**perl mergeShuffledfastqSeqs.pl -f1 left_reads.fastq -f2 right_reads.fastq -r '^@(\S+)(\s)([1|2]\S+)$' -o output_prefix -t**

## Optimisation of TopHat alignment parameters

The TopHat alignment parameters were optimised by running an alignment on one sample multiple times, where four selected parameters were adjusted each time via small increments, starting with default settings. The success of optimisation was assessed through comparison of the overall read alignment rate for each experiment conducted.

**./tophat --num-threads 6 -o output_file --read-mismatches 6 --max-deletion-length 6 --max-insertion-length 6 --read-edit-dist 6 reference_index_basename <list_left_reads.fastq> <list_right_reads.fastq> <list_unpaired_reads.fastq>**

## Variant Calling

Firstly, SAMtools was used to sort and index the ‘mapped’ binary alignment/map (BAM) files from the TopHat alignments:

**./samtools sort -o sorted_output.bam aligned_input.bam**

**./samtools index sorted_output.bam**

An mpileup file was then generated using SAMtools, as this is the required format for variant calling with VarScan. The Base Alignment Quality (BAQ) adjustment command for mpileup was disabled as recommended due to its stringency, and all other settings were set at default. Aligned BAM files from each replicate sample were provided to mpileup, which produces a multi-sample pileup format. The output was then piped to VarScan using the mpileup2* subcommands, for variant calling. The VarScan mpileup2snp command was run to call SNPs, and the mpileup2indel command was run to call indels, both with the recommended default settings, which resulted in two files listing the identified, respective variants.

**./samtools mpileup -q 1 –B –A -f reference.fasta <list_sorted_output_files.bam> | java -jar VarScan.v2.3.9.jar mpileup2snp mpilup_Liv_to_ref --min-coverage 10 --min-var-freq 0.20 --p-value 0.05 > varscan_output.snp**

**./samtools mpileup -q 1 –B –A -f reference.fasta <list_sorted_output_files.bam | java -jar VarScan.v2.3.9.jar mpileup2indel --min-coverage 10 --min-var-freq 0.10 --p-value 0.1 > varscan_output.indel**

The identified SNPs were further filtered to remove potential false positive calls. This involved executing the ‘bam-readcount’ program developed by VarScan, to obtain the necessary metrics for each SNP, followed by running VarScan’s accessory Perl script to create two output files: SNPs passing the filter and SNPs failing the filter.

**./bam-readcount –q 1 –b 20 –f reference.fasta –l varscan_output.snp sorted_output.bam > snp_readcounts**

**perl fpfilter.pl varscan_output.snp snp_readcounts --output-basename varscan_snp_filter_output**

The VarScan indel variant callset was also filtered by increasing the stringency of available parameters, to obtain a callset of higher confidence:

**./samtools mpileup -q 1 -B -f reference.fasta <list_sorted_output_files.bam> | java -jar VarScan.v2.3.9.jar mpileup2indel --min-coverage 10 --min-reads2 4 --min-var-freq 0.15 --p-value 0.05 > varscan_indel_filter**

##

## De novo transcriptome assembly

An in-house reference was created by performing a de novo transcriptome assembly using the Trinity software. The TopHat alignment tool was first used to map the *N. caninum* reads to the Vero genome, resulting in unmapped bam files consisting of only *N. caninum* reads.

**./tophat --num-threads 6 -o output_file --read-mismatches 6 --max-deletion-length 6 --max-insertion-length 6 --read-edit-dist 6 Vero_index_basename <list_left_reads.fastq> <list_right_reads.fastq> <list_unpaired_reads.fastq >**

SAMtools was then used to sort and split the unmapped bam files into paired end reads, and convert them into fastq files. The custom Perl script was again implemented to ensure paired read information was preserved, resulting in two paired read files, and an unpaired read file.

**./samtools sort -o sorted.bam unmapped.bam**

**./samtools view -h -b -f 0x40 sorted.bam > sorted_1.bam**

**./samtools view -h -b -f 0x80 sorted.bam > sorted_2.bam**

**./samtools bam2fq sorted_1.bam > sorted_1.fastq**

**./samtools bam2fq sorted_2.bam > sorted_2.fastq**

**perl mergeShuffledfastqSeqs.pl -f1 sorted_1.fastq -f2 sorted_2.fastq -r '^@(\S+)/[1|2]$' -t -o output_prefix**

These three fastq files were subsequently used to perform a de novo transcriptome assembly using the Trinity software.

**./Trinity --seqType fq --max_memory 50G --left <list_left_paired_end_reads> –right <list_right_paired_end_reads> –single <list_unpaired_reads> --CPU 6**

Redundant contigs were then arranged and resolved into clusters using CD-HIT-EST (Li and Godzik 2006). This resulted in two output files: a FASTA file containing representative sequences from each cluster, and a text file listing each cluster and the associated contigs.

**cd-hit-est -T 14 -i transcriptome.fasta -o Trinity_clusters -c 0.9 -n 5 -d 0 -g 1 -M 2000**

## Building a BLAST database to annotate variant positions

**./makeblastdb -in ToxoDB-30_NcaninumLIV_AnnotatedProteins.fasta -out Toxo_protein_db -dbtype prot**

**./blastx -db Toxo_protein_db -query <transcriptome_fasta_file> -outfmt "6 qseqid sseqid evalue bitscore length pident mismatch gaps" -out <output file>**

# Supplementary Results

## De novo transcriptome assembly with Trinity using NC-Liverpool RNA-seq data

Table S2. Metrics extracted from the Trinity de novo transcriptome assembly, following quality assessment with the Trinity and TransRate software.

| **Metric** | **Pre CD-HIT-EST** | **Post CD-HIT-EST** |
| --- | --- | --- |
| **Total number of contigs** | 50110 | 45297 |
| **Total assembled bases** | 32426361 | 27570740 |
| **Mean contig length** | 647.1 | 608.67 |
| **N50** | 883 | 807 |
| **Percentage GC** | 56.32% | 56.47% |
| **Percentage of proper pairs mapped** | 85.87% ^♦^ | 86.09% ^♦^ |

^♦^A typical Trinity transcriptome assembly will have the vast majority of all reads mapping back to the assembly, and ~70-80% of the mapped fragments found mapped as proper pairs.

## Primer Design

**Table S3. Primers designed to target variants between NC-Liverpool RNA-seq reads and the published NC-Liverpool reference genome.**

| **Chromosome** | **Variant position** | **Primer** | **Primer Sequence** | **Length (bases)** | **Product size (bp)** | **T_a_ (^o^C)** | **Variants predicted by VarScan** | **Variants confirmed by Sanger sequencing** |
| --- | --- | --- | --- | --- | --- | --- | --- | --- |
| FR823382 | 340885-340894 | Forward | TGCGTATTGGTATTTGTGTGC | 21 | 535 | 60.0 | 5 | 0 |
|  |  | Reverse | AGGAAGAGGCAAAACACGC | 19 |  |  |  |  |
| FR823384 | 1305052-1305057 | Forward | CTGGAGAAGAGACAAGAAGTTTG | 23 | 435 | 57 | 4 | 4 |
|  |  | Reverse | CAAAATACTCGAGCGTTTCC | 20 |  |  |  |  |
| FR823385 | 3870813-3870814 | Forward | TCAAGCACAGAGCGTTTCTA | 20 | 351 | 57 | 2 | 11 |
|  |  | Reverse | GTTTGATAAAGTGGACAACGTC | 22 |  |  |  |  |
| FR823389 | 1700436-1700442 | Forward | GCGAATAAGAGATAGGGTGAGA | 22 | 400 | 57 | 5 | 0 |
|  |  | Reverse | TAGGGAGAGGTAGCACGAGA | 20 |  |  |  |  |
| FR823392 | 2410585-2410594 | Forward | ACAAAGCTCCTCTGCACAATA | 21 | 386 | 57 | 6 | 6 |
|  |  | Reverse | AGAGCTGACTCCTTTGCAA | 19 |  |  |  |  |

**Table S4. Primers designed to target variants called between NC-Nowra RNA-seq reads and the de novo NC-Liverpool transcriptome.**

| **NC-Liverpool transcriptome contig ID** | **Primer** | **Primer Sequence** | **Length (bases)** | **Product size (bp)** | **T_a_ (^o^C)** | **Variants predicted by VarScan** | **Variants confirmed by Sanger sequencing** |
| --- | --- | --- | --- | --- | --- | --- | --- |
| TRINITY_DN19937_c0_g1_i1 | Forward | CCGTGTCCGTTTTCTACCG | 19 | 283 | 61 | 2 | 2 |
|  | Reverse | GGTCCAGCCCTCTTTCATG | 19 |  |  |  |  |
| TRINITY_DN25083_c0_g1_i1 | Forward | CGAACTTTGTCCAGAAGGGG | 20 | 359 | 61 | 4 | 4 |
|  | Reverse | TCGGGCTTGCGTTTTCTT | 18 |  |  |  |  |
| TRINITY_DN25501_c0_g1_i1 | Forward | TATTGATGCGTTTGGTGGC | 19 | 571 | 61 | 6 | 9 |
|  | Reverse | CATCGTTTTTCTCTGGAGTGCT | 22 |  |  |  |  |
| TRINITY_DN37292_c0_g1_i1 | Forward | TGCTCTGGGGTGAGAAGG | 18 | 735 | 61 | 8 | 8 |
|  | Reverse | GAGGGAAGAAGGCAACCAAT | 20 |  |  |  |  |
| TRINITY_DN5623_c0_g1_i2 | Forward | CTCAGGGAGGATGTCGAAGA | 20 | 320 | 61 | 3 | 3 |
|  | Reverse | AATGTCCTGATTCCTTCGCC | 20 |  |  |  |  |
| TRINITY_DN5084_c0_g1_i1 | Forward | CACCCGCAAGATCTTATACAGTC | 23 | 437 | 60 | 1 | 1 |
|  | Reverse | TATGTGCAGGTGTTTGAGTTGAG | 23 |  |  |  |  |
| TRINITY_DN5676_c0_g1_i2 | Forward | ACGAGATCGCAGACGGAT | 21 | 482 | 60 | 1 | 1 |
|  | Reverse | TGGAGTCGCTTACTATCACACC | 22 |  |  |  |  |
| TRINITY_DN6656_c0_g1_i1 | Forward | TCGAGCAGTTCCTCATCAAA | 20 | 474 | 60 | 1 | 1 |
|  | Reverse | CAGCTTTGTCTCGAATGGC | 19 |  |  |  |  |
| TRINITY_DN3730_c0_g1_i1 | Forward | GGAAGAGACAGGTGAAACAACA | 22 | 433 | 60 | 1 | 1 |
|  | Reverse | AATCGATTCATGCCTTTGTCTC | 22 |  |  |  |  |
| TRINITY_DN2186_c0_g1_i1 | Forward | GGAACATTCGGCCTACACA | 19 | 419 | 60 | 1 | 1 |
|  | Reverse | AGGCAGAATGACTAGGGGGT | 20 |  |  |  |  |

## Annotation of SNP hotspots regions

**Table S5. The genomic locations of identified SNP hotspots within 50kb windows, and the gene IDs and their annotations contained within.**

| **Chromosome** | **Region start** | **Region end** | **SNPs** | **Genes** | **Annotation** |
| --- | --- | --- | --- | --- | --- |
| FR823386  (V) | 900000 | 949999 | 21 | NCLIV_013170 | WD domain containing protein |
|  |  |  |  | NCLIV_013200 | Hypothetical protein |
|  |  |  |  | NCLIV_013210 | Hypothetical protein |
| FR823387  (VI) | 300000 | 349999 | 44 | NCLIV_015790 | Fatty acyl-CoA desaturase |
|  |  |  |  | NCLIV_015800 | Glutamic acid-rich protein |
| FR823392  (XI) | 3000000 | 3049999 | 21 | NCLIV_056620 | Hypothetical protein  (kinase activity) |
|  |  |  |  | NCLIV_056630 | Hypothetical protein  (protein binding) |
|  |  |  |  | NCLIV_056640 | WD-40 repeat containing protein |
|  |  |  |  | NCLIV_056650 | Hypothetical protein |
|  | 3050000 | 3099999 | 21 | NCLIV_056660 | Hypothetical protein |
|  |  |  |  | NCLIV_056680 | 60S ribosomal protein L6 |
|  |  |  |  | NCLIV_056700 | 26S proteasome regulatory subunit |
|  | 3150000 | 3199999 | 26 | NCLIV_056770 | Hypothetical protein |
|  |  |  |  | NCLIV_056790 | Hypothetical protein |
|  |  |  |  | NCLIV_056800 | Protein kish |
|  |  |  |  | NCLIV_056810 | Hypothetical protein  (constituent of ribosome) |
|  |  |  |  | NCLIV_056820 | 50S ribosomal protein L22 |
|  |  |  |  | NCLIV_056830 | 60S ribosomal protein L22 |
|  | 3200000 | 3249999 | 17 | NCLIV_056850 | Hypothetical protein |
|  |  |  |  | NCLIV_056860 | Hypothetical protein |
|  |  |  |  | NCLIV_056870 | Hypothetical protein |
|  |  |  |  | NCLIV_956880 | Hypothetical protein |
|  | 3250000 | 3299999 | 16 | NCLIV_056890 | Hypothetical protein |
|  |  |  |  | NCLIV_056900 | AGAP005082-PA |
|  | 3450000 | 3499999 | 18 | NCLIV_057070 | Ribosome biogenesis protein BMS1 |
|  |  |  |  | NCLIV_057080 | RCC1 precursor |
|  |  |  |  | NCLIV_057090 | Zinc finger protein |
|  |  |  |  | NCLIV_057100 | GH23000 related  (protein binding) |
|  | 3550000 | 3599999 | 26 | NCLIV_057170 | Hypothetical protein |
|  |  |  |  | NCLIV_057180 | Suppressor Mra1 superfamily |
|  |  |  |  | NCLIV_057190 | Hypothetical protein |
|  |  |  |  | NCLIV_057210 | Hypothetical protein |
|  |  |  |  | NCLIV_057220 | Hypothetical protein |
|  | 3600000 | 3649999 | 45 | NCLIV_057250 | Hypothetical protein |
|  |  |  |  | NCLIV_057260 | Hypothetical protein |
|  |  |  |  | NCLIV_057270 | Hypothetical protein |
|  |  |  |  | NCLIV_057280 | Peptidylprolyl isomerase D |
|  |  |  |  | NCLIV_057290 | Hypothetical protein |
|  |  |  |  | NCLIV_057300 | Hypothetical protein |
|  |  |  |  | NCLIV_057310 | Hypothetical protein  (protein binding) |
|  | 3650000 | 3699999 | 23 | NCLIV_057320 | Hypothetical protein |
|  |  |  |  | NCLIV_057330 | Hypothetical protein |
|  |  |  |  | NCLIV_057360 | Hypothetical protein |
|  |  |  |  | NCLIV_057380 | Hypothetical protein |
|  | 3750000 | 3799999 | 19 | NCLIV_057450 | Hypothetical protein |
|  |  |  |  | NCLIV_057460 | Transketolase central region |
|  |  |  |  | NCLIV_057470 | Hypothetical protein  (mRNA splicing) |
|  |  |  |  | NCLIV_057490 | AP-4 complex subunit sigma-1 |
|  |  |  |  | NCLIV_057500 | Dual specificity protein phosphatase |
|  | 3800000 | 3849999 | 18 | NCLIV_057510 | Hypothetical protein |
|  |  |  |  | NCLIV_057520 | Hypothetical protein |
|  |  |  |  | NCLIV_057530 | Hypothetical protein |
|  |  |  |  | NCLIV_057540 | Hypothetical protein (endopeptidase) |
|  | 3850000 | 3899999 | 19 | NCLIV_057550 | Hypothetical protein (endopeptidase) |
|  |  |  |  | NCLIV_057570 | Hypothetical protein |
|  |  |  |  | NCLIV_057580 | Hypothetical protein |
|  | 4100000 | 4149999 | 18 | NCLIV_057800 | Hypothetical protein |
|  |  |  |  | NCLIV_057820 | Protein SEY1 homolog  (GTPase activity) |
|  |  |  |  | NCLIV_057830 | Hypothetical protein  (lipid metabolic process) |
|  | 4150000 | 4199999 | 30 | NCLIV_057850 | Hypothetical protein |
|  |  |  |  | NCLIV_057860 | Prefoldin subunit 5 |
|  |  |  |  | NCLIV_057870 | Hypothetical protein (transcription initiation) |
|  |  |  |  | NCLIV_057890 | Protein-L-isoaspartate O-methyltransferase |
|  |  |  |  | NCLIV_057900 | WD domain containing protein (translation initiation factor) |
|  | 4200000 | 4249999 | 15 | NCLIV_057910 | Hypothetical protein  (translation initiation) |
|  |  |  |  | NCLIV_057920 | Hypothetical protein  (transferase activity) |
|  |  |  |  | NCLIV_057940 | Hypothetical protein  (kinase activity) |
| FR823393  (XII) | 4500000 | 4549999 | 16 | NCLIV_065940 | Hypothetical protein  (helicase activity) |
|  |  |  |  | NCLIV_065950 | ATP-binding cassette (ABC) protein |
|  | 6250000 | 6299999 | 40 | NCLIV_068420 | Hypothetical protein |
|  |  |  |  | NCLIV_068430 | Hypothetical protein |
|  |  |  |  | NCLIV_068460 | Hypothetical protein (hydrolase) |
|  |  |  |  | NCLIV_068470 | Hypothetical protein |

**Table S6. InterProScan functional annotation and location of transcripts falling within SNP hotspots**

| **Chr** | **Gene ID** | **SNPs** | **Gene name** | **Protein family** | **Homologous superfamilies** | **Domains and repeats** | **Biological process** | **Molecular function** | **Cellular component** |
| --- | --- | --- | --- | --- | --- | --- | --- | --- | --- |
| **V** | NCLIV_013170* | 12 | Putative WD40 domain-containing protein | **IPR002933**  Peptidase M20 | **IPR029055**, Nucleophile aminohydrolases, N-terminal | **IPR001680**,  WD40 repeat. | Metabolic process, GO:0008152. | Protein binding, GO:0005515 | - |
|  |  |  |  |  | **IPR015943**, WD40/YVTN repeat-like-containing domain superfamily. |  |  |  |  |
|  |  |  |  |  | **IPR036322**, WD40-repeat-containing domain superfamily. | **IPR017986**,  WD40-repeat-containing protein. |  | Hydrolase activity, GO:0016787 |  |
|  | NCLIV_013200 | 13 | Hypothetical protein | **IPR010591**  ATP11 | **IPR010591**,  ATP11 protein | - | Protein complex assembly  GO:006461 | - | Mitochondrion  GO:005739 |
|  | NCLIV_013210 | 7 | Hypothetical protein | **IPR001619**  Sec1-like protein | **IPR036045**,  Sec1-like superfamily | - | Vesicle docking in exocytosis, GO:0006904. | - | - |
|  |  |  |  |  |  |  | Vesicle-mediated transport, GO:0016192. |  |  |
| **VI** | NCLIV_015790♦ | 12 | Putative fatty acyl-CoA desaturase | **IPR015876**,  Acyl-CoA desaturase | **IPR011992**,  EF-hand domain pair | **IPR002048**,  EF-hand domain | Lipid metabolic process, GO:0006629 | Calcium ion binding, GO:0005509 | - |
|  | NCLIV_015800 | 32 | Putative glutamic acid-rich protein | - | - | - | - | - | - |
| **XI** | NCLIV_056650 | 14 | Hypothetical protein | - | - | - | - | - | - |
|  | NCLIV_056660 | 6 | Hypothetical protein | - | - | **IPR001611**,  Leucine-rich repeat | - | - | - |
|  | NCLIV_056700 | 11 | 26S proteasome regulatory subunit rpn1, related | **IPR016643**,  26S proteasome regulatory complex, non-ATPase subcomplex, Rpn1 subunit. | **IPR011989**, Armadillo (ARM) like helical. | - | Regulation of protein catabolic process, GO:0042176. | Binding, GO:0005488. | Proteasome complex, GO:0000502. |
|  |  |  |  |  | **IPR016024**, ARM-type fold. |  |  | Enzyme regulator activity, GO:0030234. |  |
|  | NCLIV_056770 | 12 | Hypothetical protein | **IPR027640**,  Kinesin-like protein. | - | - | Microtubule-based movement, GO:0007018. | Microtubule motor activity, GO:0003777. | - |
|  | NCLIV_056870 | 7 | Hypothetical protein | **IPR019155**, uncharacterised protein family | **IPR011989**, ARM-like helical. | - | - | - | - |
|  | NCLIV_056900• | 14 | AGAP005082-PA, related | **IPR026847**,  vacuolar protein sorting-associated protein 13. | - | **IPR026854**, N-terminal domain of Chorein (TM vesicle-mediated sorter. | - | - | - |
|  |  |  |  |  |  | **IPR009543**, vacuolar sorting-associated protein 13, SHR-binding domain. |  |  |  |
|  |  |  |  |  |  | **IPR031645**, vacuolar sorting-associated protein 13, C-terminal. |  |  |  |
|  | NCLIV_057080 | 6 | Regulation of chromosome condensation (RCC1) precursor | - | **IPR009091,** RCC1/BLIP-II | **IPR000408,**  RCC1 repeat | - | - | - |
|  | NCLIV_057090 | 9 | Putative zinc finger (C3HC4 type RING finger) protein | - | **IPR013083**,  Zinc finger, RING/FYVE/PHD-type. | **IPR004331**,  SPX domain. | - | Metal ion binding, GO:0046872 | - |
|  |  |  |  |  |  | **IPR001841**, Zinc finger, RING-type. |  | Protein binding, GO:0005515. |  |
|  |  |  |  |  |  | **IPR018957**,  Zinc finger, C3HC4 RING-type. |  | Zinc ion binding, GO:0008270. |  |
|  | NCLIV_057190 | 6 | Hypothetical protein | - | **IPR005612 (PF03914)**, CBF/Mak21 family | **IPR005612**, CCAAT-binding factor | - | - | - |
|  | NCLIV_057210♦ | 15 | Hypothetical protein | - | - | - | - | - | - |
|  | NCLIV_057290* | 9 | Hypothetical protein | - | - | - | - | - | - |
|  | NCLIV_057310 | 25 | Hypothetical protein | - | - | - | - | - | - |
|  | NCLIV_057320 | 14 | Hypothetical protein | - | **IPR011990**, tetratricopeptide-like helical domain superfamily. | **IPR019734**, tetratricopeptide repeat. | - | Protein binding, GO:0005515 | - |
|  | NCLIV_057390 | 6 | GTP binding protein | **-** | **IPR027417,** P-loop containing nucleoside triphosphate hydrolase | **IPR006073,** GTP binding protein | - | GTP binding, GO:0005525 | - |
|  | NCLIV_057460* | 5 | Transketolase central region | **-** | **IPR029061,** Thiamin diphosphate-binding fold | **IPR005475,** transketolase-like, pyrimidine-binding domain | Metabolic process, GO:0008152 | Catalytic activity, GO:0003824 | - |
|  |  |  |  |  | **IPR009014,** Transketolase C-terminal/pyruvate-ferredoxin oxidoreductase domain II | **IPR033248,** transketolase, C-terminal domain |  |  |  |
|  | NCLIV_057470 | 9 | Hypothetical protein | **IPR026300**,  CWF11 family | **IPR027417**,  P-loop containing nucleoside triphosphate hydrolase. | **IPR032174**,  intron-binding protein aquarius, N-terminal. | mRNA splicing, via spliceosome, GO:0000398. | - | Spliceosomal complex, GO:0005681 |
|  |  |  |  |  |  | **IPR003593**, AAA+ ATPase domain. |  |  |  |
|  | NCLIV_057510 | 6 | Hypothetical protein | - | **-** | - | - | - | - |
|  | NCLIV_057540 | 7 | Hypothetical protein | - | **IPR011989**, ARM-like helical. | - | - | Binding, GO:0005488 | - |
|  |  |  |  |  | **IPR016024**, ARM-type fold. |  |  |  |  |
|  | NCLIV_057550• | 13 | Unspecified product | **IPR015500**, Peptidase S8, subtillisin-related. | **IPR036852**, peptidase S8/S53 domain superfamily. | **IPR000209**, peptidase S8/S53 domain. | Proteolysis, GO:0006508. | Serine-type endopeptidase activity, GO:0004252. | Cytoplasm, GO:0005737. |
|  |  |  |  |  | **IPR021157**,  cytochrome c1, transmembrane anchor, C-terminal. | **IPR034204**, subtillisin SUB1-like catalytic domain. | Peptide cross-linking, GO:0018149. |  |  |
|  | NCLIV_057570* | 5 | Hypothetical protein | - | - | IPR013929, RNA polymerase II-associated protein 1, C-terminal | - | - | - |
|  | NCLIV_057800♦ | 9 | Hypothetical protein | - | - | - | - | - | - |
|  | NCLIV_057830 | 7 | Hypothetical protein | - | **IPR017946,** PLC-like phosphodiesterase, TIM beta/alpha barrel domain superfamily | - | Lipid metabolic process,  GO:0006629 | Phosphoric diester hydrolase activity,  GO:0008081 | - |
|  | NCLIV_057850 | 7 | Hypothetical protein | - | - | - | - | - | - |
|  | NCLIV_057890* | 9 | Protein-L-isoaspartate O-methyltransferase related | **IPR000682**, protein-L-isoaspartate (D-aspartate) O-methyltransferase | **IPR029063**,  S-adenosyl-L-methionine-dependent methyltransferase. |  | Cellular protein modification process, GO:0006464. | Protein-L-isoaspartate O-methyltransferase activity, GO:0004719. | - |
|  | NCLIV_057900 | 9 | Putative WD domain, G-beta repeat containing protein | - | **IPR015943**,  WD40/YVTN repeat-like-containing domain superfamily. | **IPR0017986**,  WD40-repeat-containing domain. | - | Protein binding, GO:0005515 | - |
|  |  |  |  |  | **IPR036322**, WD40-repeat-containing domain superfamily. | **IPR001680**,  WD40-repeat. |  |  |  |
|  | NCLIV_057910 | 7 | Hypothetical protein | **IPR001040**, translation initiation factor elF-4e | **IPR023398,** translation initiation factor elF-4e-like |  | Translation initiation, GO:0006413 | RNA binding, GO:0003723 | Cytoplasm, GO:0005737 |
|  |  |  |  |  |  |  |  | Translation initiation factor activity, GO:0003743 |  |
|  | NCLIV_057920* | 7 | Hypothetical protein | - | - | **-** | - | - | - |
|  | NCLIV_065950 | 14 | Putative ATP-binding cassette sub-family B member 5 | - | **IPR036640**,  ABC transporter, type 1, transmembrane domain superfamily. | **IPR011527**, ABC transporter type 1, transmembrane domain. | Transmembrane transport, GO:0055085. | ATP binding, GO:0005524. | Integral component of membrane, GO:0016021 |
| **XII** |  |  |  |  |  | **IPR003439**, ABC transporter-like. |  | ATPase activity, GO:0016887. |  |
|  |  |  |  |  | **IPR027417**,  P-loop containing nucleoside triphosphate hydrolase. | **IPR003593**, AAA+ ATPase domain. |  | ATPase activity, coupled to TM movement of substances, GO:0042626 |  |
|  | NCLIV_068460 | 12 | Unspecified product | **IPR000407**, Nucleoside phosphatase GDA1/CD39 | - | - | - | Hydrolase activity, GO:0016787 | - |
|  | NCLIV_068470 | 23 | Hypothetical protein | - | - | - | - | - | - |

* Genes containing signal peptides

♦Genes coding for transmembrane proteins.

• The blue highlighted genes were identified as transmembrane proteins containing a signal peptide.

**References**

Barber J, Trees AJ, Owen M 1993. ISOLATION OF NEOSPORA-CANINUM FROM A BRITISH DOG. Veterinary Record 133: 531-532. doi: 10.1136/vr.133.21.531

Barber JS, et al. 1995. Characterization of the first European isolate of Neospora caninum (Dubey, Carpenter, Speer, Topper and Uggla). Parasitology 111 ( Pt 5): 563-568.

Conrad PA, Barr BC, Anderson ML, Sverlow K. 2004. Recombinant neospora antigens and their uses. In. United States: The Regents of the University of California.

Conrad PA, et al. 1993. INVITRO ISOLATION AND CHARACTERIZATION OF A NEOSPORA-SP FROM ABORTED BOVINE FETUSES. Parasitology 106: 239-249. doi: 10.1017/s0031182000075065

Dubey JP, Hattel AL, Lindsay DS, Topper MJ 1988. Neonatal Neospora caninum infection in dogs: isolation of the causative agent and experimental transmission. J Am Vet Med Assoc 193: 1259-1263.

Li W, Godzik A 2006. Cd-hit: a fast program for clustering and comparing large sets of protein or nucleotide sequences. Bioinformatics 22: 1658-1659. doi: 10.1093/bioinformatics/btl158

McAllister MM, et al. 1998. Dogs are definitive hosts of Neospora caninum. Int J Parasitol 28: 1473-1478.

McInnes LM, Irwin P, Palmer DG, Ryan UM 2006. In vitro isolation and characterisation of the first canine Neospora caninum isolate in Australia. Vet Parasitol 137: 355-363. doi: 10.1016/j.vetpar.2006.01.018

Miller CM, Quinn HE, Windsor PA, Ellis JT 2002. Characterisation of the first Australian isolate of Neospora caninum from cattle. Aust Vet J 80: 620-625.

Stenlund S, Bjorkman C, Holmdahl OJM, Kindahl H, Uggla A 1997. Characterization of a Swedish bovine isolate of Neospora caninum. Parasitol Res 83: 214-219. doi: 10.1007/s004360050236

Yamane I, et al. 1996. In vitro isolation of a bovine Neospora in Japan. Veterinary Record 138: 652-652.

Yamane I, et al. 1997. In vitro isolation and characterisation of a bovine Neospora species in Japan. Res Vet Sci 63: 77-80. doi: 10.1016/s0034-5288(97)90162-4
